# Effects of Simvastatin on Cartilage Homeostasis in Steroid-Induced Osteonecrosis of Femoral Head by Inhibiting Glucocorticoid Receptor

**DOI:** 10.3390/cells11243945

**Published:** 2022-12-07

**Authors:** Yaling Yu, Lishan Lin, Kangping Liu, Yixin Jiang, Zhenlei Zhou

**Affiliations:** Department of Veterinary Clinical Science, College of Veterinary Medicine, Nanjing Agricultural University, Nanjing 210095, China

**Keywords:** steroid-induced osteonecrosis of femoral head, simvastatin, cartilage homeostasis, HIF pathway, glucocorticoid receptor

## Abstract

Steroid-induced osteonecrosis of femoral head (SONFH) is one of the most common bone disorders in humans. Statin treatment is beneficial in preventing the development of SONFH through anti-inflammation effects and inhibition of the glucocorticoid receptor (GR). However, potential mechanisms of statin action remain to be determined. In this study, pulse methylprednisolone (MP) treatment was used to induce SONFH in broilers, and then MP-treated birds were administrated with simvastatin simultaneously to investigate the changes in cartilage homeostasis. Meanwhile, chondrocytes were isolated, cultured, and treated with MP, simvastatin, or GR inhibitor in vitro. The changes in serum homeostasis factors, cell viability, and expression of GR were analyzed. The results showed that the morbidity of SONFH in the MP-treated group increased significantly compared with the simvastatin-treated and control group. Furthermore, MP treatment induced apoptosis and high-level catabolism and low-level anabolism in vitro and vivo, while simvastatin significantly decreased catabolism and slightly recovered anabolism via inhibiting GR and the hypoxia-inducible factor (HIF) pathway. The GR inhibitor or its siRNA mainly affected the catabolism of cartilage homeostasis in vitro. In conclusion, the occurrence of SONFH in broilers was related to the activation of GR and HIF pathway, and imbalance of cartilage homeostasis. Simvastatin and GR inhibitor maintained cartilage homeostasis via GR and the HIF pathway.

## 1. Introduction

Glucocorticoids (GCs) have been widely used clinically for decades as potent anti-inflammatory and immunosuppressive agents [1,2]. However, long-term or extensive administration of GCs exerts many negative effects, and steroid-induced necrosis of the femoral head (SONFH) is one of the most common complications [3]. Studies have shown that pulse GCs treatment led to a significant high incidence of SONFH during the 2003 SARS outbreak, according to epidemic investigation in China [4]. With the COVID-19 epidemic, the GCs administration has also increased significantly, so the incidence of SONFH is expected to rise [4,5]. As a progressive and destructive orthopedic disease, if SONFH is not detected and treated in time, most patients will develop femoral head collapse within 1 to 3 years [6]. Once the collapse occurs, it is difficult to reverse, and finally the patients will develop severe clinical symptoms and even disability, which can only be treated with artificial joint replacement [4]. As SONFH mainly occurs in young and middle-aged people [7], the long-term effect of artificial joint replacement is still difficult to predict. Therefore, it is crucial for early diagnosis and intervention of SONFH.

Although many pathophysiological mechanisms of SONFH have been proposed, such as impaired microcirculation, disturbed coagulation-fibrinolytic system, abnormal fat metabolism, and imbalanced differentiation of osteogenic and adipogenic factors, significant gaps remain in the understanding of SONFH pathogenesis [8]. Many studies report that impaired vascularization leads to partial ischemia and hypoxia of the femoral head, and finally leads to osteonecrosis [9]. Meanwhile, a previous study also demonstrates that imbalance of cartilage homeostasis plays a critical role in the pathogenesis of SONFH in animals [10]. Cartilage homeostasis is maintained by catabolic and anabolic factors, while the cartilage homeostasis is destroyed, the extracellular matrix (ECM) is abnormally degraded, and bone growth and development would be afflicted [11,12]. Administration of MP increases the level of catabolic factors and decreases the anabolic factors in serum [10]. Most of these cartilage homeostasis factors are regulated by the hypoxia-inducible factor (HIF) pathway, and HIF-1α activity is significantly increased in chondrocytes under the conditions of hypoxic, inflammatory, and mechanical stress [13]. Furthermore, HIF-1α is not only critical in cartilage development and homeostasis, but is also involved in anaerobic energy generation and proteoglycan synthesis of chondrocytes [13]. Many studies show that HIF-1α expression is closely involved in the progression of articular cartilage degeneration, for it is the critical transcription factor in maintaining cartilage ECM homeostasis and chondrocyte survival [14]. Meanwhile, some studies indicate that prolonged HIF-1α signaling causes skeletal dysplasia due to the limitations of synthesis energy and protein function [15].

The glucocorticoid receptor (GR) might play a critical role In the process of SONFH, as GCs regulate genes transcription via the activation of cytoplasmic GR, nuclear translocation, and binding of GC response elements [16]. Many drugs are administrated to deal with SONFH in experiments, including various traditional Chinese medicines, and lipid-lowering and anti-inflammatory drugs [17,18]. Statins, one kind of hotspot drugs used to treat bone-related diseases, are the most effective lipid-lowering drugs which act as HMG-CoA reductase inhibitors and block the conversion of HMG-CoA to mevalonate, inhibit cholesterol biosynthesis in the liver, and then inhibit the production of mevalonate isoprenoid derivatives, such as farnesyl pyrophosphate (FPP) and geranylgeranyl pyrophosphate (GGPP) [19]. As FPP could activate GR [20], we supposed that statins can interfere with SONFH via reducing the activity of GR. Furthermore, statins have anti-inflammatory effects [21], which inhibit certain inflammatory mediators, such as interleukin-1β (IL-1β), to regulate cartilage homeostasis [22,23]. Statins promote bone formation and increase bone density [24,25]. Therefore, they are often administrated for some bone disorders, including osteoarthritis, arthritis, and osteonecrosis, but there is very little in the literature about their application in SONFH [21,23,26].

In addition, most existing studies were based on the lipid-lowering function of statins, and their effects on GR and cytokines that affect cartilage homeostasis has always been ignored. In this study, a SONFH model was established with pulse MP treatment in broilers to study whether statins could regulate cartilage homeostasis via inhibiting GR and the HIF pathway, and then affect the pathogenesis of SONFH.

## 2. Materials and Methods

### 2.1. Simvastatin Treatment

As simvastatin (SMV) (Zhejiang Tianrui Pharmaceutical Co., Ltd., Zhejiang, China) is an inactive prodrug, it should hydrolyze from the lactone to the active dihydroxy-open form before administration. Briefly, SMV was hydrolyzed in NaOH/ethanol, pH 10–11, for 2 h at 50 °C, before statin solutions were neutralized with 2 N HCl [21]. The requirement dose determined the final concentrations of statins, which were dissolved in PBS (cat. P1020, Beijing Solarbio Biotechnology Co., Ltd., Beijing, China) with a pH of 7, and stored at −80 °C prior to use [27,28].

### 2.2. Animals and Treatments

#### 2.2.1. Animal Treatment and Sample Collection

All animal works were carried out in accordance with the Guidelines for Laboratory Animals of the Ministry of Science and Technology (2006, Beijing, China), and the agreement was approved by the Animal Protection and Use Committee of Nanjing Agricultural University (#NJAU-Poult-PTA2019014, approved on 21 March 2019). One-day-old broiler chickens (Gallus gallus, AA broilers) were randomly divided into 4 groups (12 chickens per group) and all birds were reared with a standardized broiler feeding process (ingredient compositions were exhibited in Supplementary Table S1). At the age of 29 d, the three experimental groups were treated with different methods of administration, as follows: the broilers in group M and MS were treated with MP for 7 days (20 mg kg^−1^ d^−1^, Haisco Pharmaceutical Group, Shenyang, China); in group MS and S, SMV was administered subcutaneously (SC) at 20 mg kg^−1^ from the day that the broilers were treated with MP to the end of the experiment (5 days every week); and birds in group C were received with an isodose sterile saline. During the whole experiment, the changes in behaviors, body weight (BW), and appetition of broilers were recorded. Half of the birds in each group were sacrificed and weighed at 42 d old and 56 d old, respectively. According to the International Cartilage Repair Society (ICRS) score, the score of cartilage damage was evaluated.

The blood samples from sacrificed broilers were collected to obtain serum and plasma, and stored at −20 °C. The femoral head was cut along the sagittal plane; half was fixed in clean 4% paraformaldehyde at 4 °C, and the other half was carefully washed with physiological saline and then stored in liquid nitrogen.

#### 2.2.2. Histopathology

After washing overnight at room temperature, the fixed cartilage tissues were decalcified in EDTA decalcifying solution (cat. G1105-500ML, Services Biotechnology Co., Ltd., Wuhan, China) for 2 weeks. After dehydration with ethanol, hyalinization with dimethylbenzene and embedment in paraffin took place. Then, the fragments of femoral head were cut into 4 μm-thick pieces and stained with eosin (H&E) for pathological observation.

#### 2.2.3. Bone Biomechanical Tests

The left bones were cleaned of all adherent tissue, and we measured the weight, length, and the diameter at the midpoint of the bones. Then, bone mineral density (BMD) was measured by a dual-energy X-ray absorption measuring instrument (Medikors, Inc., Gyeonggi-do, Korea). The test mode was set to fast scan with a high-energy parameter of 80 kVp/1.0 mA and a low-energy parameter of 55 kVp/1.25 mA. The acquired images were analyzed using the InAlyzer 1.0 image processing system to save images and analyze values.

To determine bone-breaking strength, the method of three-point flexural bending tests was carried out (LR10K PLUS, Lloyd Instruments Ltd., Hampshire, UK). Each bone sample was positioned in the middle portion of diaphysis with the same posture, which could support the maximum stability. The vertical load of 15 mm/min was applied and remained constant until fracture. The values were registered and analyzed via the machine software (NEXYGEN Plus).

#### 2.2.4. Chicken Serum and Plasma Assays

The serum was used for ELISA assay, and plasma was used for biochemical analysis. The indicators were measured according to the instructions of the ELISA kit (Nanjing Angle Gene Biotechnology Co., Ltd., Nanjing, China). The kits were chicken-specific for the detection of cartilage homeostasis factors (IL-1β (cat. ANG-E32031C), interleukin-6 (IL-6, cat. ANG-E32013C), insulin-like growth factor 1 (IGF-1, cat. ANG-E32048C) and vascular endothelial growth factor (VEGF, cat. ANG-E32043C), ECM indicators (C-telopeptide of collagen-Ⅱ (CTX-Ⅱ, cat. ANG-E32220C), procollagen Ⅱ C-propeptide (PⅡCP, cat. ANG-E32223C), col-10 (cat. E32236C) and aggrecan (cat. E32239C)) and bone metabolism indicators (alkaline phosphatase (BALP, cat. ANG-E32226C), osteocalcin (OT, cat. ANG-E32164C), tartrate acid phosphatase (TRACP-5b, cat. ANG-E32229C), and type I collagen C-terminal peptide (CTX-I, cat. ANG-E32233C)). Each sample was tested three times.

The levels of triglyceride (TG, cat. OR202, MedicalSystem Biotechnology Co., Ltd., Ningbo, China), total cholesterol (TC, cat. OR201, MedicalSystem Biotechnology Co., Ltd., Ningbo, China), high-density lipoprotein cholesterol (HDL-C, cat. OR207T, MedicalSystem Biotechnology Co., Ltd., Ningbo, China), and low-density lipoprotein cholesterol (LDL-C, cat. OR203T, MedicalSystem Biotechnology Co., Ltd., Ningbo, China) were tested by an automatic biochemical analyzer (Hitachi Ltd., Tokyo, Japan). Each sample was also measured three times.

#### 2.2.5. RNA Extraction and Real-Time Quantitative PCR

The expressions of the related genes in femoral head were detected by a quantitative real-time PCR (qRT-PCR) on the ABI PRISM 7300 HT sequence detection system (Applied Biosystems, Inc., Foster City, CA, USA). The genes selected were as follows: col-2, HIF-1α, matrix metallopeptidase 13 (MMP13), bone morphogenetic protein 2 (BMP2), VEGF, and so on. Quantitative data were normalized relative to the housekeeping GAPDH. The genes’ primer sequences as described above were listed in Supplemental Table S2. All PCR operations were performed in triplicate. The results were analyzed as relative fold change (2^−ΔΔCT^ value).

#### 2.2.6. Western Blotting Analysis

The articular cartilages were harvested by immunoblotting lysis buffer (cat. P0013B, Beyotime Biotechnology Co., Ltd., Shanghai, China) containing protease inhibitor mixture (cat. G2008-1ML, Services Biotechnology Co., Ltd., Wuhan, China). The lysates were incubated on ice for 30 min followed by centrifugation at 12,000× *g* for 20 min to remove precipitations. The supernatants were transferred into clean 1.5 mL tubes. The protein concentration in the supernatant was determined by the bicinchoninic acid (BCA) method (cat. P0012, Beyotime Biotechnology Co., Ltd., Shanghai, China). The extracts were mixed with SDS loading dye (cat. LT101S, Shanghai Epizyme Biotechnology Co., Ltd., Shanghai, China), heated at 99 °C for 10 min, before 10 ug of the protein sample was loaded into SDS-polyacrylamide gels, and they were subjected to gel electrophoresis (SDS-PAGE). Subsequently proteins were electro-transferred to polyvinylidene difluoride (PVDF) membranes (Sigma-Aldrich, Shanghai Trading Co., Ltd., Shanghai, China). The membrane was blocked with 5% (*w*/*v*) milk for 2 h and incubated with primary antibody at 4 °C overnight. The antibody information was depicted in Supplemental Table S3. After primary antibody incubation, the membrane was washed 3 times with TBST and incubated with horseradish peroxidase-conjugated anti-rabbit (1:3000) secondary antibodies (cat. S0001, Proteintech Biotechnology Co., Ltd., Wuhan, China) for 2 h. Excessive secondary antibody conjugated with HRP was then rinsed off by washing the membrane 3 times with TBST. The membrane was visualized by ECL reagents (cat. P0018AS, Beyotime Biotechnology Co., Ltd., Shanghai, China). The levels of the proteins were quantitatively analyzed and compared with GAPDH as an internal control. The Bio-Rad Gel Doc 2000 system (Bio-Rad, Hercules, CA, USA) was used to determine the gray levels of the bands.

### 2.3. Cells and Treatments

#### 2.3.1. Primary Chondrocytes Isolation, Culture, and Identification

Under strict aseptic conditions, the proximal tibial cartilages of broilers aged 4 to 6 weeks were separated and cut into small pieces. Primary chondrocytes were isolated by sequential digestion with 0.25% trypsin (cat. S310JV, Shanghai BasalMedia Biotechnology Co., Ltd., Shanghai, China) for 30 min, 0.1% hyaluronidase (cat. 9001-54-1, Sigma-Aldrich Trading Co., Ltd., Shanghai, China), and 0.2% type Ⅱ collagenase (cat. 17101015, Gibco Life Technologies, NY, USA), overnight at 37 °C and in a 5% CO_2_ incubator. The resulting crude cell suspension was passed through a sterile 70 μm cell strainer for purification. Ultimately, the chondrocytes were seeded with the density for 2 × 10^5^ cells/mL in 6 cm cell plates and high-sugar DMEM medium (cat. L110KJ, Shanghai BasalMedia Biotechnology Co., Ltd., Shanghai, China) containing 1% penicillin (cat. S110JV, Shanghai BasalMedia Biotechnology Co., Ltd., Shanghai, China) and 10% fetal bovine serum (FBS, cat. F2442S, Sigma-Aldrich Trading Co., Ltd., Shanghai, China).

The chondrocytes were dealt with Trizol (cat. RG-51001A, Nanjing Angle Gene Biotechnology Co., Ltd., Nanjing, China) to extract the total RNA. The complementary DNA (cDNA) was synthesized via reverse transcription utilizing HiScript Ⅱ QRT SuperMix (cat. R222-01, Zazyme Biotechnology Co., Ltd., Nanjing, China). Here, PCR (cat. P021-01, Zazyme Biotechnology Co., Ltd., Nanjing, China) and nucleic acid gel electrophoresis were used to detect the relative gene expression in chondrocytes. The primer sequences of these genes in this study were as follows: aggrecan, 5′-TGCAAGGCAAAGTCTTCTACG-3′ and 5′-GGCAGGGTTCAGGTAAACG-3′; collagen-2 (col-2), 5′-ACCTACAGCGTCTTGGAGGA-3′ and 5′-ATATCCACGCCAAACTCCTG-3′; col-10, 5′-ATATCCACGCCAAACTCCTG-3′ and 5′-TCAGAGGAATAGAGACCATTGGAT-3′.

#### 2.3.2. Staining of Cell

Staining is often used to identify chondrocytes. Toluidine blue staining was carried out as follows: the cells were seeded in a culture plate with cell slides. After the density of cells grew to 70%, they were washed three times with PBS and fixed with 4% paraformaldehyde for 30 min. Then, we rinsed the slides with running water for 15 min and ultra-pure water 3 times, and cells were immersed in 0.1% toluidine blue solution (cat. G2542, Solarbio Biotechnology Co., Ltd., Beijing, China) for 30 min; then, we washed away the remaining dye solutions. The pictures stained were recorded by microscopy.

Alcian blue staining was as follows: we operated as described in the instructions of the Alcian blue staining solution (cat. G1560, Solarbio Biotechnology Co., Ltd., Beijing, China).

#### 2.3.3. Cell Viability Assay and Treatment

In the present study, the second passage of chondrocytes were chosen to conduct the next experiments. Before the official test, the chondrocytes were treated to determine the final concentrations and time of MP and SMV [17,27,29]; the chondrocytes were administrated with an MP of 2 to 16 mg/mL for 15 min, and an SMV of 1, 2, 10, 25, or 50 μM for 24 or 48 h to test cell viability using a Cell Counting Kit-8 (cat. BS350A, CCK-8, Biosharp Biotechnology Co., Ltd., Hefei, China). According to the results of CCK-8 assay, the cultivated cells were divided into four groups, as follows: the first group was a negative control with conventional medium, one group was administrated with MP, an experimental group was treated with SMV, and the chondrocytes in the final group were treated with MP and SMV together.

#### 2.3.4. Apoptosis Detection

Apoptosis of chondrocytes in each group was detected by flow cytometry with annexin V-FITC/PI double staining (cat. KGA105-KGA108, KeyGEN Biotechnology Co., Ltd., Nanjing, China). Here, FITC-labeled annexin V could be used to detect phosphatidylserine on the surface of the cell membrane during apoptosis. Pyridine iodide (PI) could stain necrotic cells or cells with late apoptosis that lose cell membrane integrity. The treatment of cultured cells followed the manufacturer’s instructions.

#### 2.3.5. ELISA Assay

The cell supernatants were used to detect the level of cartilage homeostasis. The indicators were measured according to the instructions of the ELISA kit (Nanjing Angle Gene Biotechnology Co., Ltd., Nanjing, China). The kits were chicken-specific for the detection of cartilage homeostasis factors (IL-1β, IL-6, IGF-1, and VEGF). Each sample was tested three times.

#### 2.3.6. Immunocytofluorescence Analysis

When the chondrocytes grew to 70% confluence, the cells were treated with various administrations. Then, they were fixed with 4% paraformaldehyde for 15 min and permeabilized with 0.1% Triton X-100 (cat. BS084, 100 mL, Biosharp Technology Co., Ltd., Hefei, China). Cells were then blocked with 4% BSA and incubated with a primary antibody against GR (1:200, cat. WL02695, Wanleibio Co., Ltd., Shenyang, China) or HIF-1α (1:200, cat. AF7087, Beyotime Biotechnology Co., Ltd., Shanghai, China) overnight at 4 °C, and a PE-labeled secondary antibody for 1 h at 37 °C. Cells were stained with DAPI (cat. C1005, Beyotime Biotechnology, Shanghai, China) and photographed under an inverted fluorescence microscope. The chondrocytes were rinsed three times with PBS between each step.

#### 2.3.7. GR Inhibitor Treatment

The chondrocytes were seeded in 6-well plates, resulting in 70% confluence. Then, cells were administrated with different treatments, as follows: the first group was a negative control with conventional medium, one group was administrated with MP, an experimental group was treated with 100 nM GR inhibitor (RU486, cat. HY13683, MCE Biotechnology Co., Ltd., Shanghai, China) for 24 h to inhibit the expression of GR, and the chondrocytes in the final group were treated with MP and RU486 together. Finally, the RNA and protein of all groups obtained were used for following studies.

#### 2.3.8. Small Interfering RNA

The chicken GR siRNA sequences and the scrambled (NC) siRNA sequence were obtained from Genepharma Co., Ltd. (Shanghai, China). The chondrocytes were seeded in 6-well plates and kept in DMEM medium (10% FBS) without antibiotics, resulting in 70% confluence. Next, the cells were transfected with GR siRNA and scrambled siRNA using Lipofectamime 3000 transfection reagent (cat. L3000015, Thermo Fisher, Waltham, MA, USA, 2 mL/well) for 36 h in accordance with the manufacturer’s instructions. The grouping method was the same as for the RU486 grouping. Protein and mRNA samples were collected for further detection after transfection. The siRNA sequences used in this study were as follows: GR sense, 5’-UGUAUCACUAUGAUUUAAATT-3’; GR antisense: 5’-UAAAU CAUAGUGAUACAUCUG-3′.

### 2.4. Statistical Analysis

The statistical analysis was conducted using IBM SPSS Statistics 19. The morbidity of SONFH in different groups was evaluated by X^2^ tests. All other data were presented as mean ± SE, and the differences between groups were determined with one-way analysis of variance (ANOVA, Dunnet’s T3). Significant differences were defined at only one level as follows: *p* < 0.05 (significant).

## 3. Results

### 3.1. Experiment Results

#### 3.1.1. SMV Could Lower the Incidence of SONFH

The evaluation and classification criteria of femoral head necrosis in broilers was based on previous reports [12,30], and the morbidity was counted according to the number of legs instead of the number of broilers in each group. The broilers at the age of 42 d did not show lameness, while two broilers in group M exhibited an evident lameness at the age of 56 d. As for the morbidity and evaluation of SONFH, from Table 1, the MP-treated group had a higher morbidity, while the morbidity in group MS was evidently decreased.

**Figure 1:**
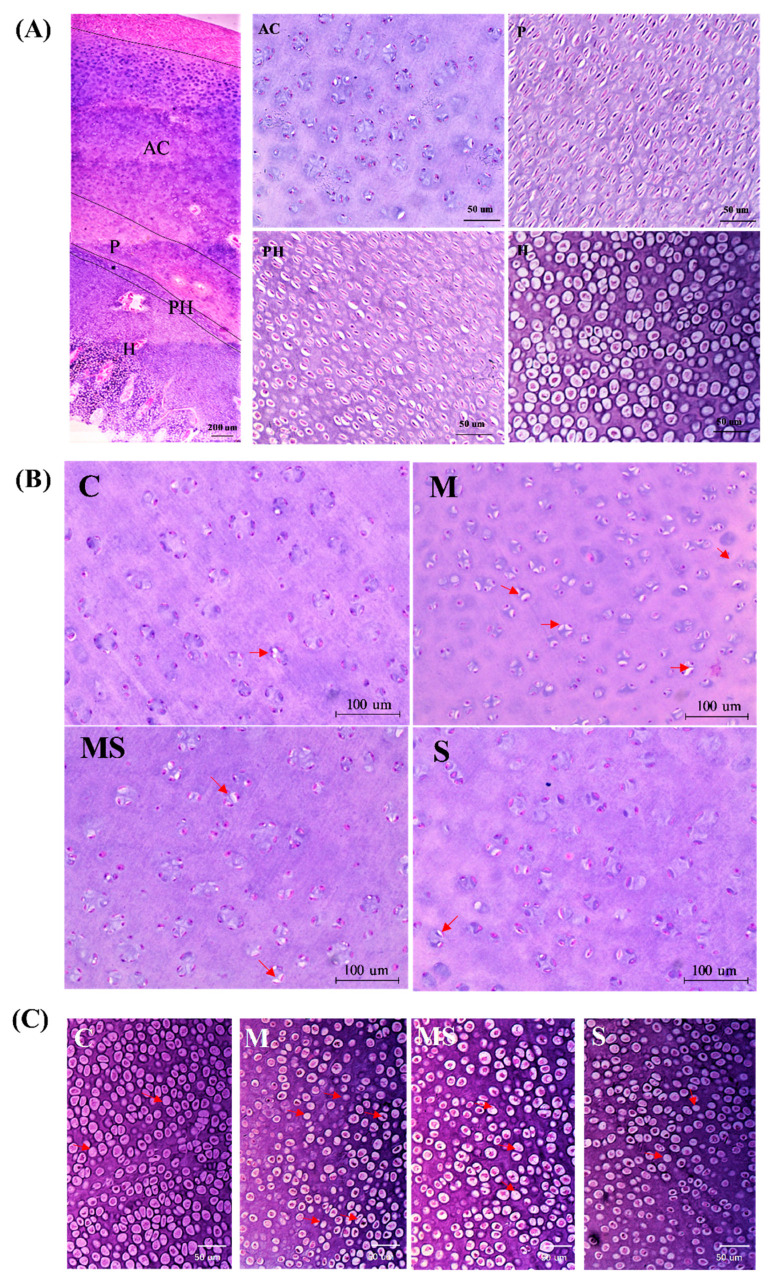

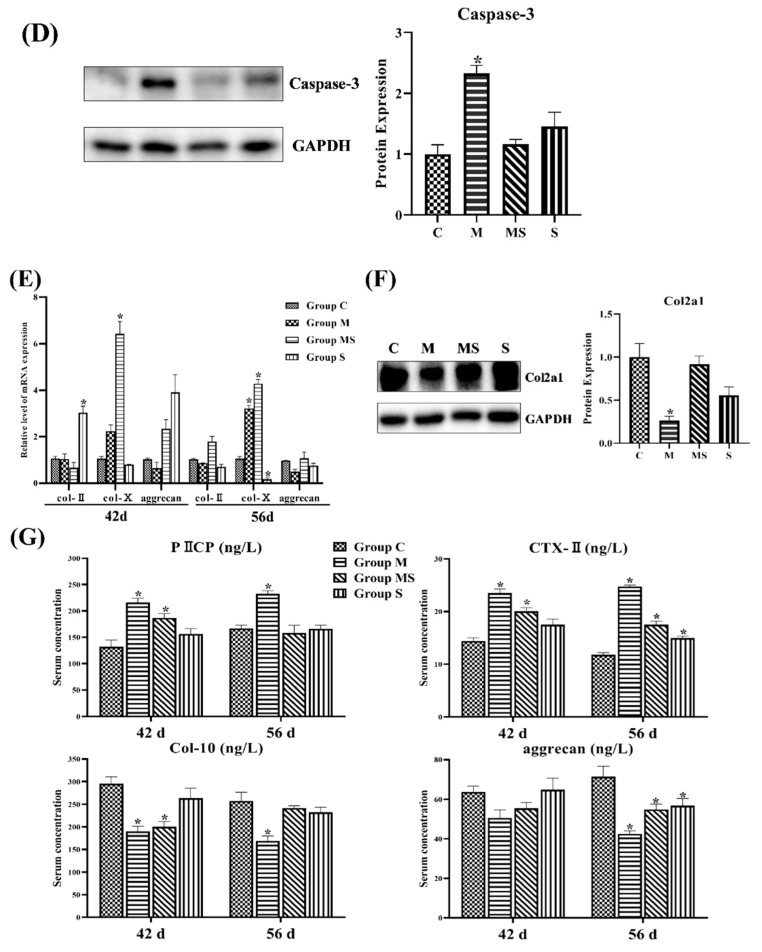
MP induced FHN in broilers. (**A**) The H&E staining of the femoral head in normal femoral head including articular cartilage (**AC**), proliferative zone (**P**), prehypertrophic zone (**PH**), and hypertrophic zone (**H**). (**B**) The H&E staining of articular cartilage in different group. The red arrow points to the chondrocytes with intracytoplasmic vacuoles and pyknosis. (**C**) The H&E staining of chondrocytes in the hypertrophic zone in different groups. (**D**) The caspase-3 expression of articular cartilage in different groups. (**E**) The 42 d and 56 d RNA expression of ECM. (**F**) The col-2a1 expression of articular cartilage in different groups. (**G**) The changes in serum collagen-Ⅱ decomposition product (CTX-Ⅱ), collagen-Ⅱ synthesis product (PⅡCP), collagen-Ⅹ, and aggrecan in different groups. * *p* < 0.05 vs. control group.

The ICRS score analysis (Supplementary Figure S1) also showed that MP treatment caused cartilage damage in the femoral head, and SMV rescued this damage (Table 2). Meanwhile, MP caused more intracytoplasmic vacuoles and pyknosis in the articular cartilage and hypertrophic zone of the femoral head, and the expression of caspase-3 was elevated (Figure 1). The related indicators (BW, feed conversion, and heart, spleen and liver index) showed that the physiological state was only slightly affected (Supplementary Figure S2). Cartilage of the femoral head was composed of chondrocytes and ECM that contained col-2, col-10, aggrecan, and so on [31]. Our data showed that there were high expressions of CTX-Ⅱ and PⅡCP (CTX-II and PIICP were produced during the decomposition and synthesis of col-2, respectively) in group M, and the protein level of col-2 was significantly lower. As for col-10 and aggrecan, they were significantly reduced in MP-treated broilers. In co-treated broilers, SMV alleviated ECM degradation.

**Figure 2:**
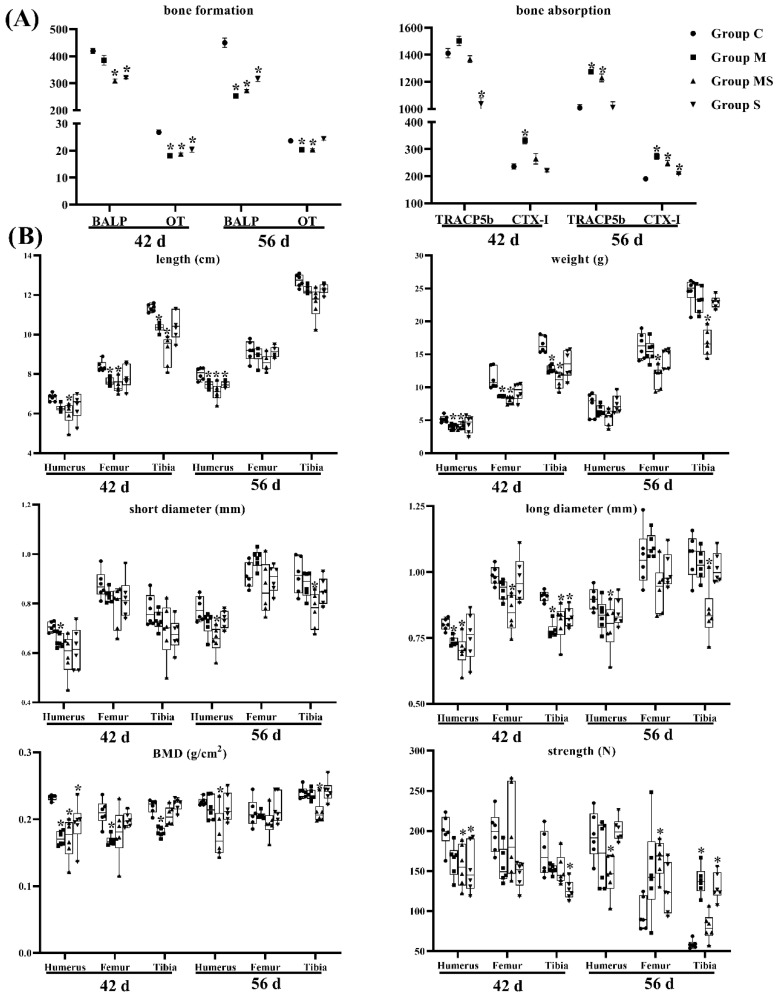
The changes in bone remodeling in broilers (*n* = 6). (**A**) The changes in serum indicators related with bone metabolism in broilers. (**B**) The changes in bone parameters in broilers. Here, * *p* < 0.05 vs. the control group.

On the other hand, the M group had low-level bone formation indicators (BALP and OT) and high-content bone resorption indicators (TRACP5b and CTX-Ⅰ). Bone was in a constant state of remodeling between formation and resorption [20], and most bone diseases could be attributed to excessive resorption [32]. We found that bone quality in group M was in poor condition (Figure 2). The bone biomechanical indicators also confirmed the finding. The MS group showed that the SMV slightly mitigated the impacts of MP on bone metabolism, but did not exhibit protection on bone biomechanics.

#### 3.1.2. MP Did Not Affect Lipid Metabolism

**Figure 3:**
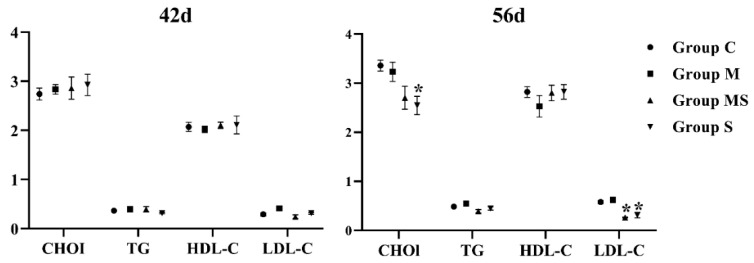
The changes in indicators related with lipid metabolism in broilers. Here, * *p* < 0.05 vs. control group.

The etiology of SONFH was complex, and abnormal lipid metabolism was one of the well-accepted factors [33]. Figure 3 showed that the levels of TG, TC, and LDL-C were promoted, and that HDL-C was inhibited in the M group without significance, while these impacts were mitigated in the MS group. Therefore, MP might not affect lipid metabolism in a short time.

#### 3.1.3. SMV Mitigated the Imbalance of Homeostasis Induced by MP via Inhibiting HIF Pathway

Here, ECM was regulated by homeostasis-related factors in cartilage, which contain catabolic and anabolic factors, related to ECM degradation and generation, respectively [34]. Furthermore, VEGF, IGF1, and BMP2 were anabolic factors that maintained ECM homeostasis in explanted articular cartilage [35]. Here, IL was an inflammatory factor and the most important catabolic factor, which was involved in the degradation of collagen in cartilage along with MMP13 and MMP2 [36]. Therefore, this study detected the level of cartilage homeostasis factors.

**Figure 4:**
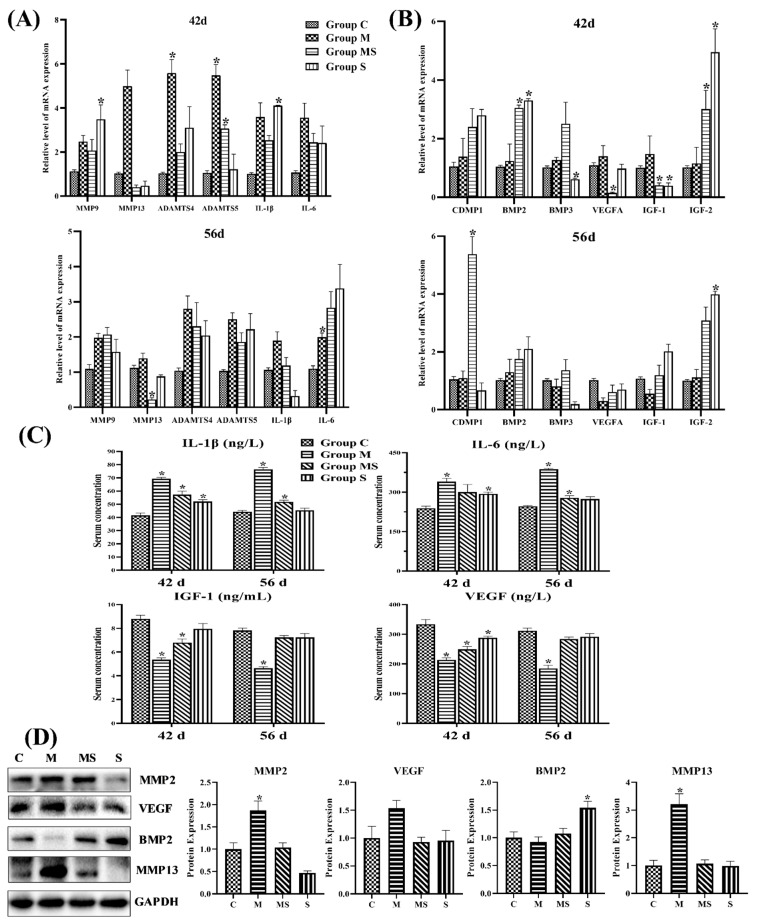

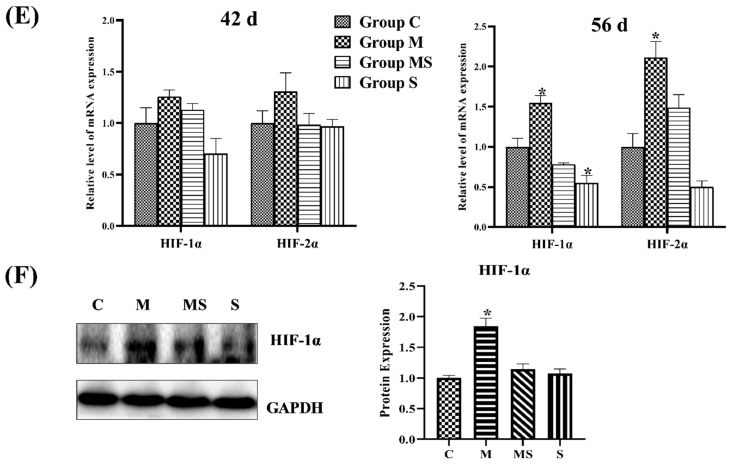
The changes in cartilage homeostasis and the HIF pathway in articular cartilage. (**A**) The 42 d and 56 d RNA expression of catabolic related genes in articular cartilage. (**B**) The 42 d and 56 d RNA expression of anabolic related genes in articular cartilage. (**C**) The changes in serum catabolic cytokines (IL-1β and IL-6) and anabolic cytokines (VEGF and IGF-1) in four groups at the age of 42 d and 56 d. (**D**) The 56 d protein expression of homeostasis-related factors in articular cartilage. (**E**) The 42 d and 56 d mRNA expressions of HIF pathway in articular cartilage. (**F**) The 56 d protein expressions of HIF-1α in articular cartilage. Here, * *p* < 0.05 vs. control group.

As shown in Figure 4, the mRNA and protein levels of catabolism significantly increased, whereas anabolism decreased with slight significance in SONFH cartilage. Based on the results, the catabolism played a major role in the process of SONFH. Meanwhile, subjects co-treated with SMV attempted to restore homeostasis, as homeostasis factors in broilers co-treated with MP and SMV recovered to normal; MMP13 and BMP2 exhibited especially significant changes.

The VEGF, MMP2, and MMP13 are the target genes of the HIF pathway [36]. Meanwhile, the HIF pathway played a critical role in promoting the expression of cartilage-related genes and regulating energy metabolism and ECM synthesis in chondrocytes. Figure 4E showed that MP activated the HIF pathway, while SMV significantly lowered the expressions of HIF-1α in cartilage. The results might predict that the high-level HIF pathway disrupted ECM homeostasis by promoting catabolism.

#### 3.1.4. SMV Mitigated the ECM Degradation and Apoptosis in MP-Treated Chondrocytes

**Figure 5:**
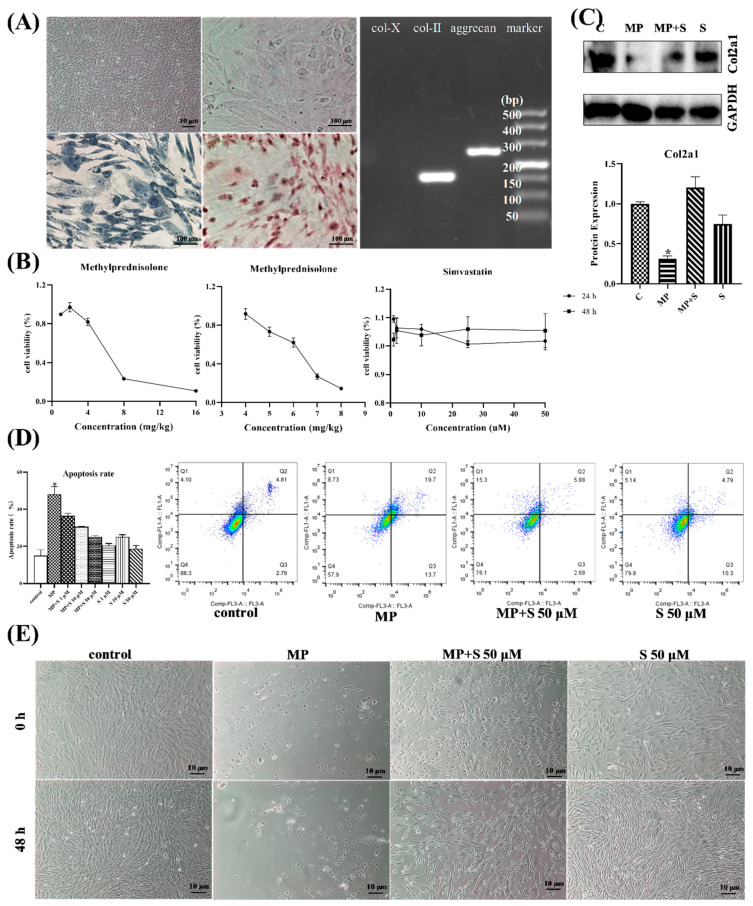
The MP-treated chondrocytes showed ECM degradation and apoptosis. (**A**) The chondrocytes with toluidine blue staining of chondrocytes (200×) or alcian blue staining of chondrocytes (200×), and the results of touchdown PCR from chondrocytes. (**B**) The viability of chondrocytes under different concentrations of MP or simvastatin. (**C**) The col-2a1 expression of chondrocytes with different administrations. (**D**) The results of apoptosis of cells in different groups by flow cytometry determination. (**E**) The results of chondrocytes with different treatments. (40×). Here, * *p* < 0.05 vs. control group.

From Figure 5A, the cells showed that the paving stone was grown, and it was able to be stained with alcian blue and toluidine blue. The results of nucleic acid gel electrophoresis also showed that isolated chondrocytes could express col-2 and aggrecan successfully, while they did not express col-10. These findings demonstrated that the cells used in this work were chondrocytes. From the result of the CCK-8 assay (Figure 5B), it was appropriate that chondrocytes were treated with 6 mg/mL MP for 15 min (cell viability = 62.02%) and 50 μM SMV for 48 h (there were no differences between treatment groups) in the present study.

As the most important collagen, col-2 could represent the level of ECM [37], and we found that MP induced ECM degradation in chondrocytes. Moreover, it was evident that MP also induced severe cell death (Figure 5E) and apoptosis (14.97 to 48.07%) (Figure 5D) in chondrocytes, while the co-treated group showed that SMV evidently protected chondrocytes from MP treatment in a dose- and time-dependent manner. In addition, the chondrocytes treated with SMV alone did not exhibit visible protections from apoptosis. The staining of alcian blue and toluidine blue showed similar results (Supplementary Figure S3).

#### 3.1.5. SMV Inhibited the HIF Pathway to Restore Cartilage Homeostasis In Vitro

**Figure 6:**
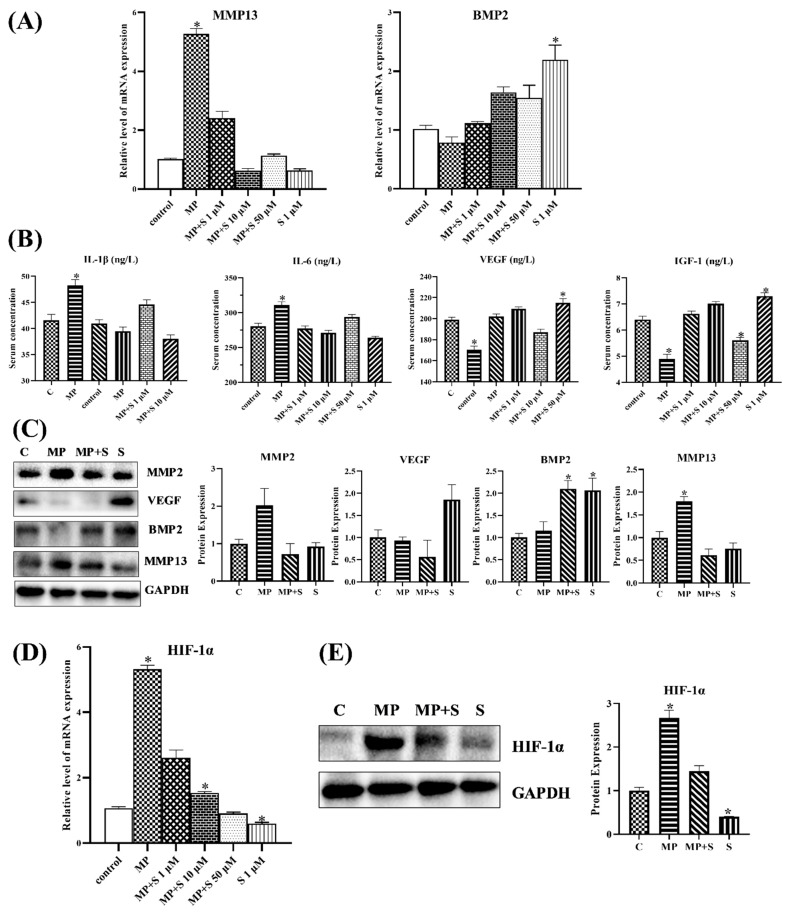
The changes in cartilage homeostasis and the HIF pathway in chondrocytes. (**A**) The mRNA expression of BMP2 and MMP13 in chondrocytes. (**B**) The changes in catabolic cytokines and anabolic cytokines in cell supernatant with different treatments. (**C**) The protein expression of homeostasis-related factors in chondrocytes. (**D**) The mRNA expressions of the HIF pathway in chondrocytes. (**E**) The protein expressions of HIF-1α in chondrocytes. Here, * *p* < 0.05 vs. control group.

The homeostasis-related factors (catabolic and anabolic factors) in cartilage could be generated by the chondrocytes [34] and then regulate the ECM metabolism and life activities in chondrocytes. The results of vitro experiments showed that the transcription level of catabolic factors increased significantly, whereas anabolic factors slightly decreased in MP-treated chondrocytes (Figure 6A). Meanwhile, the chondrocytes co-treated with SMV attempted to restore homeostasis, and catabolism and anabolism returned to normal. The findings of related proteins further verified that the cartilage homeostasis in MP-treated chondrocytes was out of balance. The ELISA and western blotting analysis of MP-treated chondrocytes suggested that the catabolic factors related to ECM degradation, such as IL-1β, IL-6, MMP2, and MMP13, were promoted, and that the anabolic factors related to ECM production, including VEGF, IGF1 and BMP2, were inhibited (Figure 6B,C). As in the results of the mRNA expressions, SMV mitigated the effects of MP on cartilage homeostasis.

As for the HIF pathway related to homeostasis, it was evidently promoted in MP-treated chondrocytes, while the chondrocytes that were co-treated with SMV showed a significant drop. The results were highly similar to the in vivo experiments. At the same time, catabolism with a more pronounced change in chondrocytes was closely linked to the activated HIF pathway.

#### 3.1.6. SMV Restrained the Expression of GR

**Figure 7:**
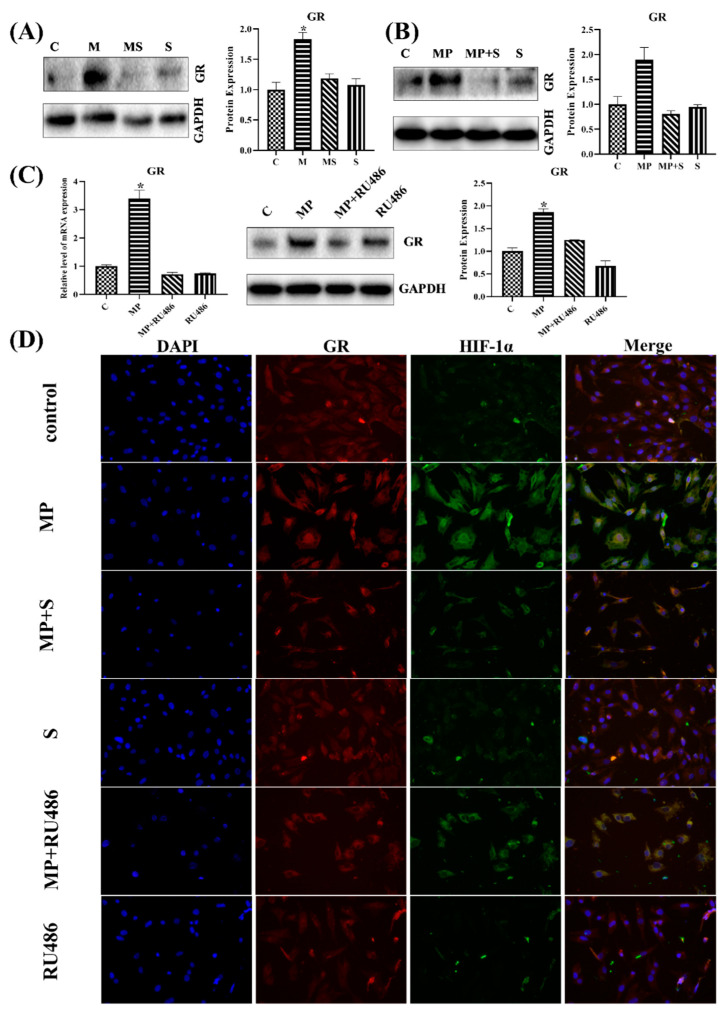
The GR expressions with different treatments. (**A**) The 56 d protein expressions of GR in articular cartilage. (**B**) The protein expressions of GR in chondrocytes. (**C**) The mRNA and protein expressions of GR in chondrocytes with RU486-treatment. (**D**) Immunocytofluorescence staining of GR (red) and HIF-1α (green) in chondrocytes with different treatments (400×). Here, * *p* < 0.05 vs. control group.

As reported in some of the literatures, GC could promote HIF-1α stabilization through GR [38], and SMV could repress GR via inhibiting the content of FPP; therefore, the expression of GR was detected in vitro and in vivo. As in those reports, the MP-treated broilers and chondrocytes activated GR, while those co-treated with SMV restored their expression to the normal level (Figure 7A–D). To confirm the role of GR in MP-induced SONFH, we attempted to use RU486, one kind of GR inhibitor, to administrate the chondrocytes. Evidently, RU486 indeed decreased the mRNA and protein level of GR and HIF-1α in MP-treated chondrocytes (Figure 7C,D).

#### 3.1.7. GR Expression Effected the Level of HIF Pathway and Homeostasis in Chondrocytes

**Figure 8:**
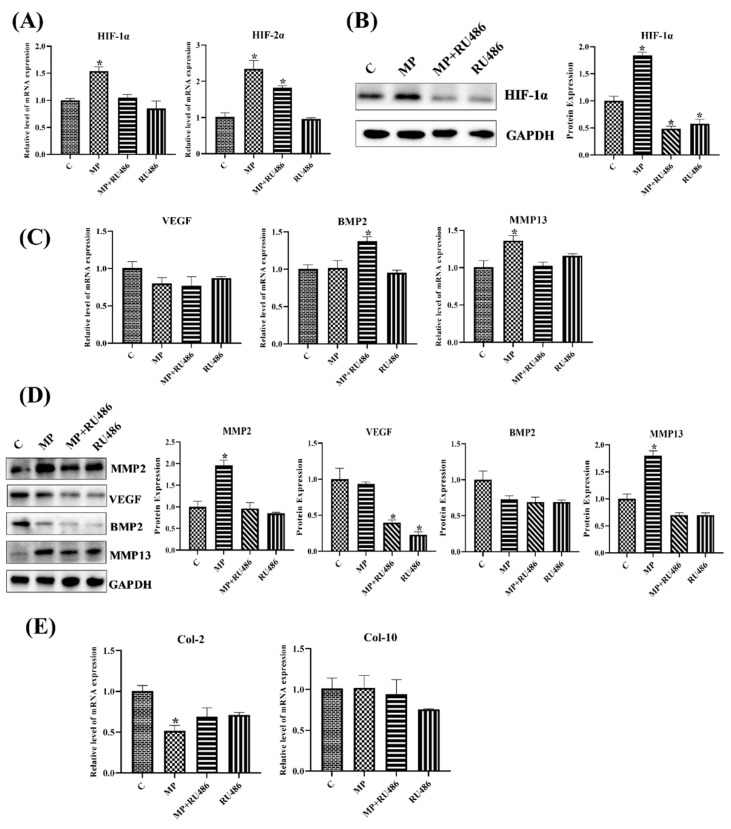
The expressions of related factors in chondrocytes with RU486-treatment. (**A**) The mRNA expressions of the HIF pathway in chondrocytes. (**B**) The protein expressions of HIF-1α in chondrocytes. (**C**) The mRNA expressions of homeostasis-related genes in chondrocytes with different treatments. (**D**) The protein expressions of homeostasis-related factors in chondrocytes. (**E**) The mRNA expressions of ECM in chondrocytes. Here, * *p* < 0.05 vs. control group.

**Figure 9:**
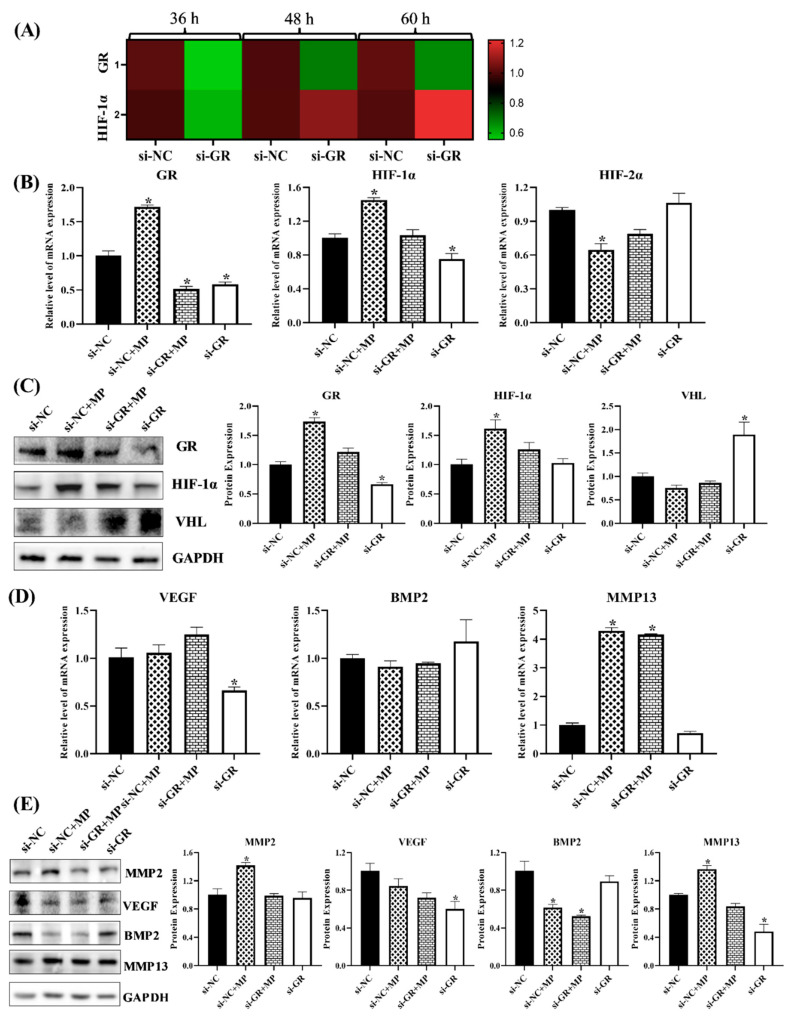

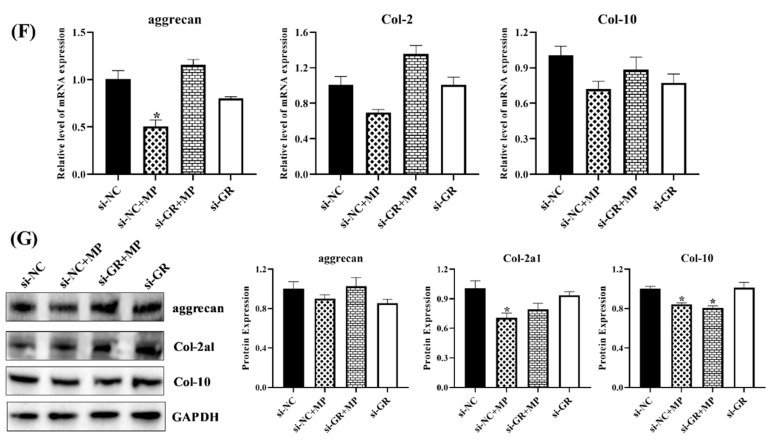
The expressions of related factors in chondrocytes with siGR-treatment. (**A**) The mRNA expressions of GR and HIF-1α in chondrocytes. (**B**) The mRNA expressions of GR and the HIF pathway in chondrocytes. (**C**) The protein expressions of GR, HIF-1α, and VHL in chondrocytes. (**D**) The mRNA expressions of homeostasis-related genes in chondrocytes with different treatments. (**E**) The protein expressions of homeostasis-related factors in chondrocytes. (**F**) The mRNA expressions of ECM in chondrocytes. (**G**) The protein expressions of ECM in chondrocytes. Here, * *p* < 0.05 vs. control group.

Due to the functions of GR in GCs, the roles of GR in the HIF pathway and homeostasis was worth exploring; therefore, the related genes and proteins were detected. The transcription and protein level of HIF-1α both showed that RU486 could repress the HIF pathway, and it verified that MP activated the HIF pathway through combining with GR (Figure 7D and Figure 8A,B). Meanwhile, the results of chondrocytes treated with GR siRNA showed similar results, the level of GR affected the expression of HIF-1α (Figure 9A), and the low-level HIF-1α was related to the activated von Hippel–Lindau tumor suppressor protein (VHL) (Figure 9B,C).

The MMP2, MMP13 and VEGF were the targets of the HIF pathway [36], and they decreased in RU486- or GR siRNA-treated chondrocytes as well (Figure 8C,D and Figure 9D,E), which further verified that there was a close relationship between GR and the HIF pathway. The MMP2 and MMP13 were catabolic factors and had similar changes as in SMV-treated chondrocytes, but VEGF, as the anabolic factor, exhibited an opposite change.

Moreover, the catabolism related to cartilage homeostasis attempted to restore itself under inhibited GR, and ECM also showed similar trends (Figure 8E). Although anabolism did not recover to normal, the ECM expression significantly increased (Figure 9E,F), suggesting that catabolism acted as a key in chondrocyte and cartilage damage caused by GCs.

## 4. Discussion

The results evidently showed that GR played a critical role in MP-indued SONFH and chondrocytes breaking. In this study, the changes in expression in GR, the HIF pathway, homeostasis factors, and ECM were detected. The activated GR could further activate the HIF pathway and its target genes. In the chondrocytes exposed to MP, the expression of GR was upregulated, and the HIF pathway was activated. The highly-expressed target genes of the HIF pathway (MMP2, MMP13, and IL-6) induced ECM degradation. Meanwhile, SMV could inhibit GR via lowering FPP [20], and then restored cartilage homeostasis and reduced the morbidity of SONFH by inhibiting the overexpression of the HIF pathway.

Chickens are bipedal animals and carry BW with two legs, and they have an intact pituitary and adrenal system as in mammals, which offers an inherent advantage when studying bone disorders in human beings [39,40,41,42]. Therefore, the broiler was used to study SONFH as a model. The results showed that MP treatment caused SONFH and apoptosis of chondrocytes in this work [3], while SMV protected broilers and chondrocytes from the impacts of MP treatment in a dose- and time-dependent manner. From the results of morbidity and evaluation of SONFH, pulse MP treatment could elevate the morbidity of SONFH, and co-treatment with SMV evidently decreased the morbidity. The SONFH broilers had imbalanced synthesis and degradation of cartilage ECM and intracytoplasmic vacuoles and pyknosis in cartilage of the femoral head [12]. The MP-treated chondrocytes and cartilages also exhibited various damages, not only apoptosis promotion and proliferation inhibition, but also a significant reduction in the expression of col-2, the main component of the ECM. These findings indicated that the chondrocyte apoptosis and disorders of ECM metabolism were common outcomes of MP-induced SONFH. Moreover, co-treated SMV evidently mitigated these impacts from MP treatment in broilers, especially balanced ECM synthesis and degradation in SONFH-affected birds, and maintained ECM expression and chondrocyte life activities in MP-treated chondrocytes.

On the other hand, SONFH showed reduced bone formation and increased bone resorption, and subjects co-treated with SMV slightly mitigated these impacts, which indicated that SMV had a limit benefit for bone remodeling. Some studies found that homeostasis factors could affect bone metabolic indicators [43]. The bone indicator results exhibited only a slightly similar trend to the homeostasis factor. Therefore, SMV not only affected bone formation indirectly by inhibiting inflammation, but had other pathways to affect bone quality [25]. For example, various concentrations of statins showed different effects on small G protein, thereby promoting or inhibiting osteogenesis [20]. In the present study, the broilers that had lower bone metabolism markers showed a poor bone biomechanics. The SMV treatment alone seemed to be ineffective for bone remodeling instead of being beneficial, which was the same as in some clinical and animal studies that supported the ineffectiveness of SMV therapy on bone tissues [44], although other findings demonstrated that statins had a bone-protective function [20,25]. The result of lower bone parameters in broilers treated with both MP and simvastatin proved our finding further. In a previous study, Frank showed that although it had early anti-inflammatory effects, the bone-protective effects of SMV did not occur until much later (Day 29) [27]; as our administration lasted only 28 days, insufficient administration time might be an alternative reason why simvastatin did not exhibit bone protection.

The synthesis and degradation of ECM could be regulated by homeostasis factors, while catabolic and anabolic factors also control chondrocyte functions [11]. Therefore, cartilage homeostasis might be vital in ECM metabolism and chondrocytes life activities. The MMP2, MMP13, IL-6, and VEGF are the target genes of the HIF pathway [38], and these genes are involved in cartilage homeostasis. The VEGF is involved in angiogenesis, and vascularization of the growth plate is necessary for chondrocyte proliferation and differentiation. The IGF1 stimulated chondrocytes proliferation and col-2 generation, while BMP2 maintained proteoglycan synthesis in explanted articular cartilage [35]. As the most important catabolic factor, IL plays multiple roles at the same time; it stimulates chondrocytes to produce proteases, such as MMPs, to promote ECM degradation, induces chondrocyte apoptosis, mediates chondrocyte hypertrophy, inhibits proteoglycan synthesis, suppresses the proliferation of chondrocytes, and so on. As a collagenase, MMP13 degrades col-I and col-II, while MMP2 mainly degrades gelatin, col-IV, and col-V [36]. In this study, the determination of homeostasis factors suggested that there was a clear homeostasis imbalance in SONFH and MP-treated chondrocytes, that appeared to be closely related to the degradation of ECM, chondrocytes destruction, and OSNFH occurrence. Moreover, the catabolic factors, such as MMP2, MMP13, and IL-6, demonstrated significant changes, whereas anabolic factor VEGF slightly recovered, which seemed to indicate that catabolism played a major role in the imbalance of homeostasis induced by MP [12]. In SMV-treated broilers and chondrocytes, both catabolism and anabolism recovered to the normal level. This further verified the idea that imbalanced homeostasis could affect ECM metabolism, and even induce SONFH and chondrocyte destruction.

The GCs are an important class of hormones which usually exert their effects through GR in vivo; GCs also have a close interplay with the HIF pathway in physiology and disease [45,46]. In fact, although the GC-GR complex was known for its anti-inflammatory effects, the mechanism was more complex. Studies had shown that loss of GR function could inhibit the transcriptional activity of factors associated with inflammation (such as cytokines IL-6, IL-1β, IL-8, and MMP13), confirming the dual effects of GC-GR on the immune system [47,48,49]. This conclusion was also confirmed in this study. As expected, MP activated GR and the HIF pathway, and the increased downstream target genes (MMP2, MMP13, and IL-6) ruined cartilage homeostasis. On the other hand, the SMV-treated chondrocytes and broilers showed lower levels of GR and HIF-1α, which appeared to be beneficial in restoring cartilage homeostasis. Based on the findings, RU486 (GR inhibitor) or GR siRNA was administrated with the chondrocytes, and the results showed that the expressions of GR and HIF-1α were similar as SMV-treated group. Not only was the transcription level of HIF-1α decreased, but, also, the protein level of VHL significantly increased. The PHD3-VHL-E3 ubiquitin ligase complex was the main regulatory mechanism for the expression of the HIF pathway, and this complex strictly controlled the low level of HIF under normoxia [50]. The VHL was one of the core components, and the high level often represents the degradation of HIF-α. However, anabolic factors in RU486- or GR siRNA-treated chondrocytes exhibited opposite changes, while catabolic factors showed similar changes to the SMV-treated chondrocytes. The findings further verified that the HIF pathway activated by GR is mainly involved in catabolism related to cartilage homeostasis instead of anabolism. The results indicated that there were other pathways involved in regulating anabolism. Combined with the ECM results, SMV would mitigate catabolism to restore MP-destroyed cartilage homeostasis via inhibiting GR and the HIF pathway, and then recovered through the expression of ECM. As for anabolism, the findings indicated that it was only slightly affected by the HIF pathway and GR.

Statins were administrated as the effective drugs for lipid-lowering, and many studies suggested that the role of lipid metabolism in SONFH cannot be ignored [10]. However, in this study, there was no significant steatosis in liver and spleen (results not shown) and no visible changes in serum lipid metabolism of MP-treated broilers, which indicated that MP might not induce SONFH by affecting lipid metabolism. Although SMV regulated fat metabolism in vivo, it was irrelative with SONFH. Considering the homeostasis-related factors, the anti-inflammatory potency did not correlate with their cholesterol-lowering efficacy [51].

**Figure 10:**
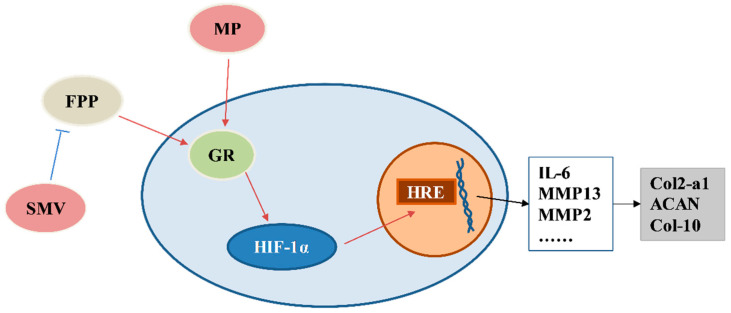
A graphic molecular mechanism of MP-treatment to regulate chondrocyte ECM metabolism.

The relationship between the pulse MP treatment and SONFH in humans has been well identified [52], and SONFH is hard to cure with medicines because of the poor self-regeneration capacity of cartilage [31]; therefore, it is vital to interfere with the occurrence and development of SONFH. As results show that the roles of GR and the HIF pathway in MP-induced SONFH and chondrocyte disruption seem to be essential (Figure 10), it is worth considering how to apply GR inhibitors to prevent the occurrence of SONFH. Moreover, the catabolism seems to be more critical than anabolism, so it is also worth noting whether catabolism is controlled at normal level when using GCs.

## 5. Conclusions

The occurrence and development of SONFH is closely related to activated GR and the HIF pathway, which could impact the catabolism factors and destroy ECM homeostasis. We found that the articular cartilage and hypertrophic zone of the femoral head in SONFH affected-broilers demonstrated visible intracytoplasmic vacuoles and pyknosis, the ECM metabolism was out of balance, and bone biomechanics were disordered. The analysis of serum samples also showed that homeostasis-related factors and bone remodeling indicators changed significantly. These might be used as indicators for early diagnosis. Meanwhile, in the present study, it was verified that SMV had anti-inflammatory effects, and further revealed that SMV could significantly decrease the catabolism and slightly recover anabolism via inhibiting GR and the HIF pathway. The GR inhibitor or its siRNA further verifies that low-level GR could suppress the HIF pathway and its downstream target genes expressions to regulate catabolism instead of anabolism. The findings not only provide a pathway to intervene in the development of SONFH, but also shed light on the molecular mechanism of MP on chondrocyte function.

## Figures and Tables

**Table 1:** FHN evaluation and morbidity in broilers, when both legs of each broiler were counted.

Group (*n* = 6)		FHN Evaluation Score			Total Morbidity (%)	Total Score	Amount
		0	1	2			
42 Day	Group C	9	2	1	25.00	4	12
	Group M	5	4	3	58.33	10	12
	Group MS	8	2	2	33.33	6	12
	Group S	10	2	0	16.67	2	12
56 Day	Group C	10	2	0	16.67	2	12
	Group M	6	3	3	50.00	9	12
	Group MS	8	3	1	33.33	5	12
	Group S	9	3	0	25.00	3	12

**Table 2:** The numbers of broilers with each gait score.

ICRS Score	Group C (*n* = 6)	Group M (*n* = 6)	Group MS (*n* = 6)	Group S (*n* = 6)
Grade 0	4	1	3	3
Grade 1	6	3	4	7
Grade 2	2	4	4	1
Grade 3	0	1	1	1
Grade 4	0	3	0	0

Note—there are five levels of cartilage damage, as follows: Grade 0, normal cartilage; Grade 1, nearly normal, with superficial lesions, soft indentation, and/or superficial fissures and cracks; grade 2, abnormal, with lesions extending down to <50% of cartilage depth; Grade 3, severely abnormal, with cartilage defects extending down >50% of cartilage depth as well as down to the calcified layer and down to but not through the subchondral bone; Grade 4, severely abnormal, with cartilage defects extending down to the subchondral bone.

## Data Availability

The datasets used and/or analyzed during the current study are available from the corresponding author on reasonable request.

## Supplementary Materials

The following supporting information can be downloaded at: https://www.mdpi.com/article/10.3390/cells11243945/s1, Supplementary materials can be found in Table S1 (Ingredient compositions and the nutrient levels of elemental diets), Table S2 (Sequences of primers used to amplify specific mRNAs by qRT-PCR), Table S3 (The information of antibodies was used in this study), Figure S1 (The femoral head cartilage with different ICRS score), Figure S2 (The physiological results of each experimental group), and Figure S3 (The toluidine blue staining and alcian blue staining of chondrocytes with different treatments).

## Abbreviations

ADAMTS, a disintegrin-like metalloprotease; BALP, alkaline phosphatase; BMD, bone mineral density; BMP, bone morphogenetic protein; BW, body weight; CDMP, cartilage morphogenetic protein; CTX-I, cross-linked carboxy-terminal telopeptide of type I collagen; CTX-Ⅱ, C-telopeptide of collagen-Ⅱ; ECM, extracellular matrix; FPP, farnesyl pyrophosphate; GC, glucocorticoid; GGPP, geranylgeranyl pyrophosphate; GR, glucocorticoid receptor; H&E, hematoxylin and eosin; HDL-C, high-density lipoprotein cholesterol; HIF, hypoxia-inducible factor; IGF, insulin-like growth factor; IL-1β, interleukin-1β; IL-6, interleukin-6; LDL-C, low-density lipoprotein cholesterol; MMP, matrix proteinase; MP, methylprednisolone; OT, osteocalcin; PⅡCP, procollagen Ⅱ C-propeptide; PBS, phosphate buffered saline; TC, total cholesterol; TG, triglyceride; TRACP-5b, tartrate acid phosphatase-5b; VEGF, vascular endothelial growth factor; VHL, von Hippel–Lindau tumor suppressor protein; SONFH, steroid-induced osteonecrosis of femoral head; SMV, simvastatin.

## References

[1] Yao X., Yu S., Jing X., Guo J., Sun K., Guo F., Sun K., Guo F., Ye Y. (2020). PTEN inhibitor VO-OHpic attenuates GC-associated endothelial progenitor cell dysfunction and osteonecrosis of the femoral head via activating Nrf2 signaling and inhibiting mitochondrial apoptosis pathway. Stem Cell Res. Ther..

[2] Marston S.B., Gillingham K., Bailey R.F., Cheng E.Y. (2002). Osteonecrosis of the femoral head after solid organ transplantation—A prospective study. J. Bone Jt. Surg. Am. Vol..

[3] Weinstein R.S., Hogan E.A., Borrelli M.J., Liachenko S., O’Brien C.A., Manolagas S.C. (2017). The Pathophysiological Sequence of Glucocorticoid-Induced Osteonecrosis of the Femoral Head in Male Mice. Endocrinology.

[4] Fu W.M., Liu B.Y., Wang B.J., Zhao D.W. (2019). Early diagnosis and treatment of steroid-induced osteonecrosis of the femoral head. Int. Orthop..

[5] Yu R.G., Zhang J.Y., Zhuo Y.G., Hong X., Ye J., Tang S.S., Liu N., Zhang Y. (2021). ARG2, MAP4K5 and TSTA3 as Diagnostic Markers of Steroid-Induced Osteonecrosis of the Femoral Head and Their Correlation with Immune Infiltration. Front. Genet..

[6] Mont M.A., Jones L.C., Hungerford D.S. (2006). Nontraumatic osteonecrosis of the femoral head, ten years later. J. Bone Jt. Surg. Am..

[7] Mont M.A., Hungerford D.S. (1995). Non-traumatic avascular necrosis of the femoral head. J. Bone Jt. Surg. Am..

[8] Yue J., Yu H., Liu P., Wen P., Zhang H., Guo W., Zhang Q. (2021). Preliminary study of icariin indicating prevention of steroid-induced osteonecrosis of femoral head by regulating abnormal expression of miRNA-335 and protecting the functions of bone microvascular endothelial cells in rats. Gene.

[9] Huang C., Wen Z., Niu J., Lin S., Wang W. (2021). Steroid-Induced Osteonecrosis of the Femoral Head: Novel Insight Into the Roles of Bone Endothelial Cells in Pathogenesis and Treatment. Front. Cell Dev. Biol..

[10] Packialakshmi B., Liyanage R., Lay J.J., Okimoto R., Rath N. (2015). Prednisolone-induced predisposition to femoral head separation and the accompanying plasma protein changes in chickens. Biomark Insights.

[11] Sandell L.J., Aigner T. (2001). Articular cartilage and changes in arthritis. An introduction: Cell biology of osteoarthritis. Arthritis Res..

[12] Yu Y., Wang S., Zhou Z. (2020). Cartilage Homeostasis Affects Femoral Head Necrosis Induced by Methylprednisolone in Broilers. Int. J. Mol. Sci..

[13] Pfander D., Swoboda B., Cramer T. (2006). The role of HIF-1alpha in maintaining cartilage homeostasis and during the pathogenesis of osteoarthritis. Arthritis Res. Ther..

[14] Yudoh K., Nakamura H., Masuko-Hongo K., Kato T., Nishioka K. (2005). Catabolic stress induces expression of hypoxia-inducible factor (HIF)-1 alpha in articular chondrocytes: Involvement of HIF-1 alpha in the pathogenesis of osteoarthritis. Arthritis Res. Ther..

[15] Stegen S., Laperre K., Eelen G., Rinaldi G., Fraisl P., Torrekens S., van Looveren R., Loopmans S., Bultynck G., Vinckier S. (2019). HIF-1α metabolically controls collagen synthesis and modification in chondrocytes. Nature.

[16] Becker P.B., Gloss B., Schmid W., Strahle U., Schutz G. (1986). In vivo protein-DNA interactions in a glucocorticoid response element require the presence of the hormone. Nature.

[17] Li H., Meng D., Zhang X., Yuan D. (2019). Effect of psoralen on the expression of PPARgamma, osteocalcin, and trabecular bone area in rabbits with steroid-induced avascular necrosis of the femoral head. J. Orthop. Surg. Res..

[18] Wu J., Yao L., Wang B., Liu Z., Ma K. (2016). Tao-Hong-Si-Wu Decoction ameliorates steroid-induced avascular necrosis of the femoral head by regulating the HIF-1alpha pathway and cell apoptosis. Biosci. Trends..

[19] Leung B.P., Sattar N., Crilly A., Prach M., McCarey D.W., Payne H., Madhok R., Campbell C., Gracie J.A., Liew F.W. (2003). A novel anti-inflammatory role for simvastatin in inflammatory arthritis. J. Immunol..

[20] Ruan F., Zheng Q., Wang J. (2012). Mechanisms of bone anabolism regulated by statins. Biosci. Rep..

[21] Palmer G., Chobaz V., Talabot-Ayer D., Taylor S., So A., Gabay C., Busso N. (2004). Assessment of the efficacy of different statins in murine collagen-induced arthritis. Arthritis Rheum..

[22] Wang Y., Tonkin A., Jones G., Hill C., Ding C., Wluka A.E., Forbes A., Cicuttini F.M. (2015). Does statin use have a disease modifying effect in symptomatic knee osteoarthritis? Study protocol for a randomised controlled trial. Trials.

[23] Yudoh K., Karasawa R. (2010). Statin prevents chondrocyte aging and degeneration of articular cartilage in osteoarthritis (OA). Aging.

[24] Soares E.A., Novaes R.D., Nakagaki W.R., Fernandes G.J., Garcia J.A., Camilli J.A. (2015). Metabolic and structural bone disturbances induced by hyperlipidic diet in mice treated with simvastatin. Int. J. Exp. Pathol..

[25] Edwards C.J., Spector T.D. (2002). Statins as modulators of bone formation. Arthritis Res..

[26] Akasaki Y., Matsuda S., Nakayama K., Fukagawa S., Miura H., Iwamoto Y. (2009). Mevastatin reduces cartilage degradation in rabbit experimental osteoarthritis through inhibition of synovial inflammation. Osteoarthr. Cartilage..

[27] Funk J.L., Chen J., Downey K.J., Clark R.A. (2008). Bone protective effect of simvastatin in experimental arthritis. J. Rheumatol..

[28] Jadhav S.B., Jain G.K. (2006). Statins and osteoporosis: New role for old drugs. J. Pharm. Pharmacol..

[29] Lazzerini P.E., Capecchi P.L., Selvi E., Lorenzini S., Bisogno S., Baldari C.T., Galeazzi M., Leigh-Pasini F. (2011). Statins and the joint: Multiple targets for a global protection?. Semin. Arthritis Rheum..

[30] Okazaki S., Nishitani Y., Nagoya S., Kaya M., Yamashita T., Matsumoto H. (2009). Femoral head osteonecrosis can be caused by disruption of the systemic immune response via the toll-like receptor 4 signalling pathway. Rheumatology.

[31] Demoor M., Ollitrault D., Gomez-Leduc T., Bouyoucef M., Hervieu M., Fabre H., Lafont J., Denoix J.-M., Audigie F., Mallein-Gerin F. (2014). Cartilage tissue engineering: Molecular control of chondrocyte differentiation for proper cartilage matrix reconstruction. Biochim. Biophys. Acta.

[32] Boyle W.J., Simonet W.S., Lacey D.L. (2003). Osteoclast differentiation and activation. Nature.

[33] Erken H.Y., Ofluoglu O., Aktas M., Topal C., Yildiz M. (2012). Effect of pentoxifylline on histopathological changes in steroid-induced osteonecrosis of femoral head: Experimental study in chicken. Int. Orthop..

[34] Reddi A.H. (2003). Cartilage morphogenetic proteins: Role in joint development, homoeostasis, and regeneration. Ann. Rheum. Dis..

[35] Kwon H., Paschos N.K., Hu J.C., Athanasiou K. (2016). Articular cartilage tissue engineering: The role of signaling molecules. Cell Mol. Life Sci..

[36] Saito T., Fukai A., Mabuchi A., Ikeda T., Yano F., Ohba S., Nishida N., Akune T., Yoshimura N., Nakagawa T. (2010). Transcriptional regulation of endochondral ossification by HIF-2alpha during skeletal growth and osteoarthritis development. Nat. Med..

[37] Sophia F.A., Bedi A., Rodeo S.A. (2009). The basic science of articular cartilage: Structure, composition, and function. Sports Health.

[38] Vettori A., Greenald D., Wilson G.K., Peron M., Facchinello N., Markham E., Sinnakaruppan M., Matthews L.C., McKeating J.A., Argenton F. (2017). Glucocorticoids promote Von Hippel Lindau degradation and Hif-1alpha stabilization. Proc. Natl. Acad. Sci. USA.

[39] Cui Q., Wang G.J., Su C.C., Balian G. (1997). The Otto Aufranc Award. Lovastatin prevents steroid induced adipogenesis and osteonecrosis. Clin. Orthop. Relat. Res..

[40] Wang G.J., Cui Q., Balian G. (2000). The Nicolas Andry award. The pathogenesis and prevention of steroid-induced osteonecrosis. Clin. Orthop. Relat. Res..

[41] Cook M.E. (2000). Skeletal deformities and their causes: Introduction. Poult. Sci..

[42] Xu J., Wang X., Toney C.B., Seamon J., Cui Q. (2010). Blood supply to the chicken femoral head. Comp. Med..

[43] Matzelle M.M., Shaw A.T., Baum R., Maeda Y., Li J., Karmakar S., Manning C.A., Walsh N.C., Rosen V., Gravallese E.M. (2016). Inflammation in arthritis induces expression of BMP3, an inhibitor of bone formation. Scand. J. Rheumatol..

[44] Hsia J., Morse M., Levin V. (2002). Effect of simvastatin on bone markers in osteopenic women, a placebo-controlled, dose-ranging trial. BMC Musculoskelet. Disord..

[45] Tokudome S., Sano M., Shinmura K., Matsuhashi T., Morizane S., Moriyama H., Tamaki K., Hayashida K., Nakanishi H., Yoshikawa N. (2009). Glucocorticoid protects rodent hearts from ischemia/reperfusion injury by activating lipocalin-type prostaglandin D synthase-derived PGD2 biosynthesis. J. Clin. Investig..

[46] Dardzinski B.J., Smith S.L., Towfighi J., Williams G.D., Vannucci R.C., Smith M.B. (2000). Increased plasma beta-hydroxybutyrate, preserved cerebral energy metabolism, and amelioration of brain damage during neonatal hypoxia ischemia with dexamethasone pretreatment. Pediatr. Res..

[47] FaFacchinello N., Skobo T., Meneghetti G., Colletti E., Dinarello A., Tiso N., Costa R., Gioacchini G., Carnevali O., Argenton F. (2017). nr3c1 null mutant zebrafish are viable and reveal DNA-binding-independent activities of the glucocorticoid receptor. Sci. Rep..

[48] Busillo J.M., Cidlowski J.A. (2013). The five Rs of glucocorticoid action during inflammation: Ready, reinforce, repress, resolve, and restore. Trends Endocrinol. Metab..

[49] Xie Y., Tolmeijer S., Oskam J.M., Tonkens T., Meijer A.H., Schaaf M.J. (2019). Glucocorticoids inhibit macrophage differentiation towards a pro-inflammatory phenotype upon wounding without affecting their migration. Dis. Model Mech..

[50] Marchi D., van Eeden F.J. (2021). Homeostatic Regulation of Glucocorticoid Receptor Activity by Hypoxia-Inducible Factor 1: From Physiology to Clinic. Cells.

[51] Arnaud C., Braunersreuther V., Mach F. (2005). Toward Immunomodulatory and Anti-Inflammatory Properties of Statins. Trends Cardiovasc. Med..

[52] Lemoine A. (1957). Vascular changes after interference with the blood flow of the femoral head of the rabbit. J. Bone Jt. Surg. Br..

