# Supplementary material for: Effects of Simvastatin on Cartilage Homeostasis in Steroid-Induced Osteonecrosis of Femoral Head by Inhibiting Glucocorticoid Receptor
Source: Cells. 2022 Dec 7;11(24):3945. doi: 10.3390/cells11243945 (PMC9777187; doi:10.3390/cells11243945)
Supplement: Supplementary file 1 [file cells-11-03945-s001.zip › cells-1983544-supplementary.pdf]

Supplement Table S1 Ingredient compositions and the nutrient levels of elemental diets (dry matter basis)

Item	Pre-feed (from 1d to 21d)	Late feed (from 22d to 56d)
**Ingredients (%)**		
Corn	57.00	62.00
Soybean meal	32.60	28.00
Corn gluten meal	3.00	2.00
Soybean oil	3.00	4.00
CaHPO4	2.00	1.60
Limestone	1.23	1.30
L-Lys·HCl	0.32	0.31
NaCl	0.30	0.30
DL-Met	0.15	0.11
Premix*	0.40	0.38
Total	100.00	100.00
**Nutrient levels[2] (%)**		
Metabolizable Energy (MJ/kg)	12.55	13.13
Crude Protein	21.10	19.57
Available Ca	1.00	0.93
Available P	0.46	0.39
Lysine	1.20	1.05
Methionine	0.50	0.42

Note: *Per kilogram of Premix containing 12 000 IU Vitamin A, 3 000 IU Vitamin D3, 30 IU Vitamin E, 1.3 mg Vitamin K3, 0.013 mg Vitamin B12, 400 mg choline chloride, 40mg niacin, 10 mg calcium pantothenate, 8 mg riboflavin, 4 mg pyridoxine, 2.2 mg thiamine, 1 mg folic acid, 0.04 mg biotin, 110 mg manganese, 80 mg iron, 65 mg Zinc, 7.5 mg copper, 1.1 mg iodine, 0.3 mg selenium.

Supplement Table S2 Sequences of primers used to amplify specific mRNAs by qRT-PCR.

Target gene	Primer sequence (5'-3')
GAPDH	Forward: GAACATCATCCCAGCGTCCA
	Reverse: CGGCAGGTCAGGTCAACAAC
MMP9	Forward: GCCATCACTGAGATCAATGGAG
	Reverse: GATAGAGAAGGCGCCCTGAGT
MMP13	Forward: AGAGACCCTGGAGCACTGATGT
	Reverse: GGGATCTCTGTCTCCAGCACCA
ADAMTS4	Forward: CAGTGGGCAGTGCGTGGATATG
	Reverse: AAGCGGTGCGTGGAGGTGAT
ADAMTS5	Forward: CGTGGTGAAGGTGGTGGTCTTG
	Reverse: GTTGTGCTGGTGCTGCCACTT
IL-1β	Forward: GGTCAACATCGCCACCTACA
	Reverse: CATACGAGATGCAAACCAGCAA
IL-6	Forward: TTCACCGTGTGCGAGAACAGC
	Reverse: CTGGAGAGCTTCGTCAGGCATT
BMP2	Forward: TGGTGGAGGTGGTTCACTTGGA
	Reverse: TCCCTTGCCATCATGCCCAAAC
BMP3	Forward: CTGACATTGGCTGGAGCGAGTG
	Reverse: TCTGGATGGTGGCATGGTTGGA
CDMP1	Forward: CGCCTCCAACTTGCTGTGTCC
	Reverse: TCCACCACCATGTCCTCGTACTG
VEGFA	Forward: GAGTTGTCGAAGGCTGCTCC
	Reverse: GCAACCCGCACATCTCATCA
IGF-1	Forward: GTATGTGGAGACAGAGGCTTC
	Reverse: TTTGGCATATCAGTGTGGCGC
IGF-2	Forward: CAACCGTGGCATTGTGGAGGAG
	Reverse: CGCTCTGACTTGACGGACTTGG

HIF-1α	Forward: CAGCCAGGTGCCGAAGAAGC<br>Reverse: ATGGTCAGCCTCATAATGGATGCC
HIF-2α	Forward: CTGTTGACGATGAGCAGTGCCT<br>Reverse: CCAGGTGTTGGAGCCAGTTGTG
collagen-Ⅱ	Forward: ACCTACAGCGTCTTGGAGGA<br>Reverse: ATATCCACGCCAAACTCCTG
collagen-Ⅹ	Forward: GCCTTCCAGGTCAGCCAGGTAT<br>Reverse: TTGCCGATGCCAACTTCTCCAG
aggrecan	Forward: TGCAAGGCAAAGTCTTCTACG<br>Reverse: GGCAGGGTTCAGGTAAACG

Supplement Table S3 The information of antibodies was used in this study.

**Antibody name**	**Company**	**Dilution**	**Catalog number**
anti-col-2a1	Beyotime	1:500	AF6528
anti-aggrecan	Beyotime	1:500	AF6126
anti-col-10	Beyotime	1:500	AF6538
anti-GAPDH	Proteintech	1:1000	AF7021
anti-HIF1α	Beyotime	1:500	AF7087
anti-GR	Wanlei	1:500	WL02695
anti-MMP2	Wanlei	1:500	WL03224
anti-VEGF	Beyotime	1:500	AF0312
anti-BMP2	Beyotime	1:500	AF0075
anti-MMP13	Wanlei	1:500	WL04694
anti-VHL	Beyotime	1:500	AF8328

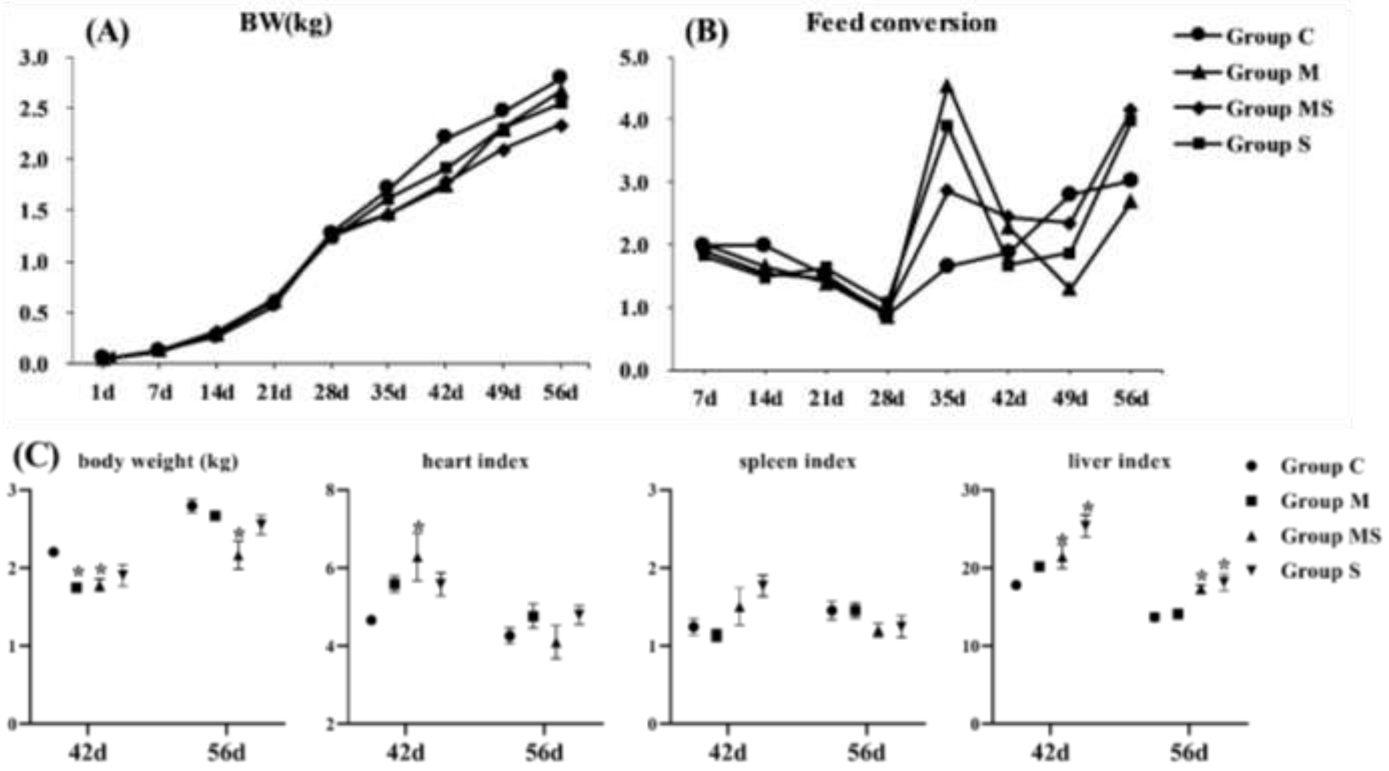

Supplement Figure S1 The physiological results of each experimental group. (A): The change of BW from day 1 to 56. (B): The trend of feed conversion at each week from day 7 to 56. (C): The changes of body weight, heart index, spleen index and liver index at the age of 42d and 56d. *, $p < 0.05$ vs control group.

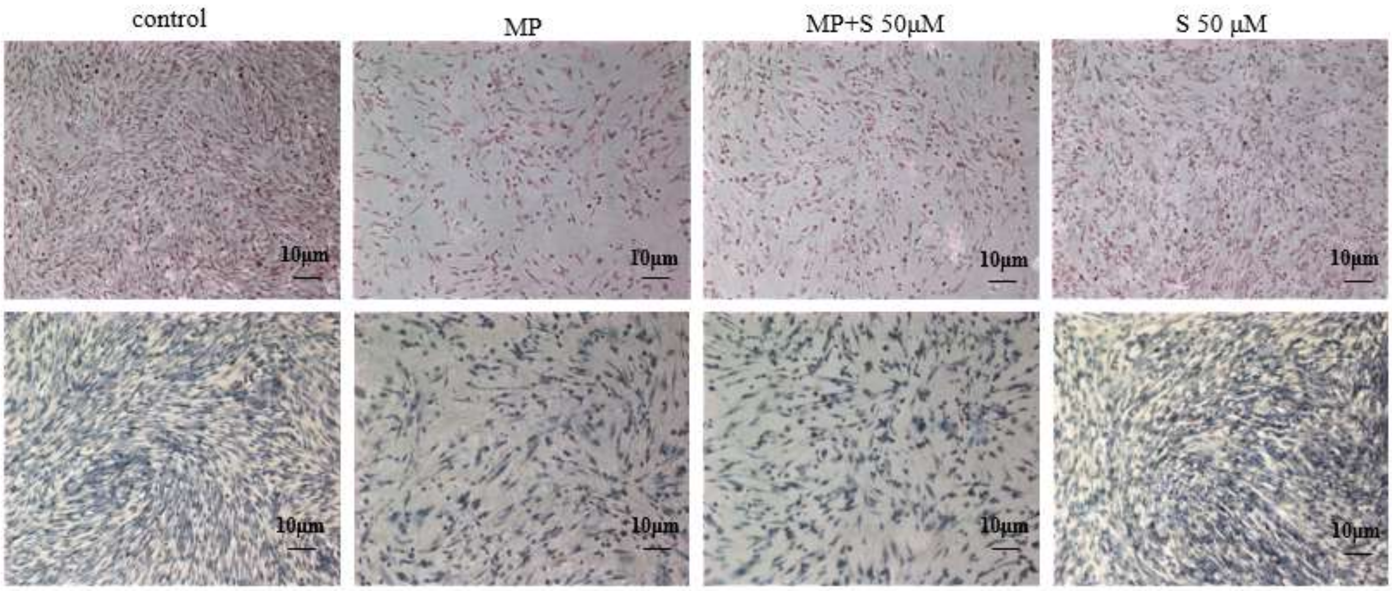

Supplement Figure S2 The toluidine blue staining and alcian blue staining of chondrocytes with different treatments.
